# 2-Amino­pyridinium (2-amino­pyridine)trichloridonickelate(II)

**DOI:** 10.1107/S1600536808005655

**Published:** 2008-03-05

**Authors:** Hoong-Kun Fun, S. Franklin, Samuel Robinson Jebas, T. Balasubramanian

**Affiliations:** a X-ray Crystallography Unit, School of Physics, Universiti Sains Malaysia, 11800 USM, Penang, Malaysia; b Department of Physics, National Institute of Technology, Tiruchirappalli 620 015, India

## Abstract

In the title compound, (C_5_H_7_N_2_)[NiCl_3_(C_5_H_6_N_2_)], the Ni^II^ atom is four-coordinated by three chloride anions and one N atom of a 2-amino­pyridine ligand, forming a distorted tetra­hedral coordination. In the crystal structure, cations and complex anions are linked into chains along the *a*, *b* and *c* axes by N—H⋯Cl hydrogen bonds, leading to the formation of a three-dimensional framework.

## Related literature

For related literature, see: Batsanov & Howard (2001 ▶); Bis & Zaworotko (2005 ▶); Chao *et al.* (1975 ▶); Corain *et al.* (1985 ▶); Jebas *et al.* (2006 ▶); Valdés-Martínez *et al.* (2001 ▶); Sletten & Kovacs (1993 ▶); Smith *et al.* (2000 ▶, 2001 ▶); Stibrany *et al.* (2004 ▶); Wei & Willett (1995 ▶); Windholz (1976 ▶).
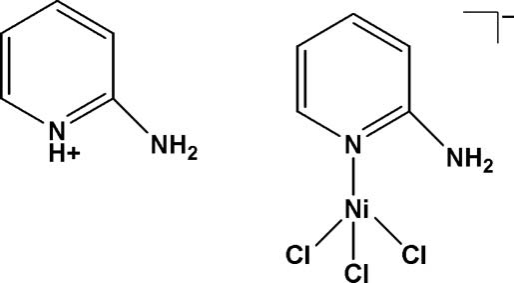


## Experimental

### 

#### Crystal data

(C_5_H_7_N_2_)[NiCl_3_(C_5_H_6_N_2_)]
*M* *_r_* = 354.3
Monoclinic, 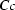

*a* = 12.9265 (1) Å
*b* = 8.0644 (1) Å
*c* = 13.9893 (1) Å
β = 106.163 (1)°
*V* = 1400.67 (2) Å^3^
*Z* = 4
Mo *K*α radiation
μ = 1.94 mm^−1^
*T* = 100.0 (1) K
0.37 × 0.08 × 0.07 mm

#### Data collection

Bruker SMART APEXII CCD area-detector diffractometer with Oxford Cryosystems Cobra low-temperature attachment
Absorption correction: multi-scan (*SADABS*; Bruker, 2005 ▶) *T*_min_ = 0.533, *T*_max_ = 0.876
19539 measured reflections
6427 independent reflections
5088 reflections with *I* > 2σ(*I*)
*R*_int_ = 0.031

#### Refinement

*R*[*F* ^2^ > 2σ(*F* ^2^)] = 0.035
*wR*(*F* ^2^) = 0.079
*S* = 1.05
6427 reflections
167 parameters
2 restraints
H atoms treated by a mixture of independent and constrained refinement
Δρ_max_ = 0.52 e Å^−3^
Δρ_min_ = −0.64 e Å^−3^
Absolute structure: Flack (1983 ▶), 1953 Friedel pairs
Flack parameter: 0.065 (9)

### 

Data collection: *APEX2* (Bruker, 2005 ▶); cell refinement: *APEX2*; data reduction: *SAINT* (Bruker, 2005 ▶); program(s) used to solve structure: *SHELXTL* (Sheldrick, 2008 ▶); program(s) used to refine structure: *SHELXTL*; molecular graphics: *SHELXTL*; software used to prepare material for publication: *SHELXTL* and *PLATON* (Spek, 2003 ▶).

## Supplementary Material

Crystal structure: contains datablocks global, I. DOI: 10.1107/S1600536808005655/ci2566sup1.cif

Structure factors: contains datablocks I. DOI: 10.1107/S1600536808005655/ci2566Isup2.hkl

Additional supplementary materials:  crystallographic information; 3D view; checkCIF report

## Figures and Tables

**Table:** 

Ni1—N1	2.0287 (17)
Ni1—Cl2	2.2625 (6)
Ni1—Cl1	2.2665 (5)
Ni1—Cl3	2.2722 (6)

**Table:** 

N1—Ni1—Cl2	114.10 (5)
N1—Ni1—Cl1	109.21 (5)
Cl2—Ni1—Cl1	107.77 (2)
N1—Ni1—Cl3	104.63 (5)
Cl2—Ni1—Cl3	108.62 (2)
Cl1—Ni1—Cl3	112.60 (2)

**Table 2:** Hydrogen-bond geometry (Å, °)

*D*—H⋯*A*	*D*—H	H⋯*A*	*D*⋯*A*	*D*—H⋯*A*
N3—H1*N*3⋯Cl2^i^	0.82 (3)	2.81 (3)	3.380 (2)	128 (2)
N2—H2*B*⋯Cl2	0.86	2.53	3.3475 (19)	159
N2—H2*C*⋯Cl1^ii^	0.86	2.63	3.4866 (19)	172
N4—H4*B*⋯Cl3^i^	0.86	2.36	3.197 (2)	165
N4—H4*C*⋯Cl1^iii^	0.86	2.54	3.344 (2)	156

Symmetry codes: (i) 
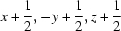
; (ii) 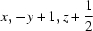; (iii) 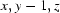.

**Fig. 1.**
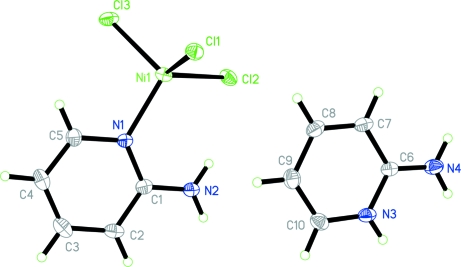
The asymmetric unit of the title compound, showing 50% probability displacement ellipsoids and the atomic numbering.

**Fig. 2.**
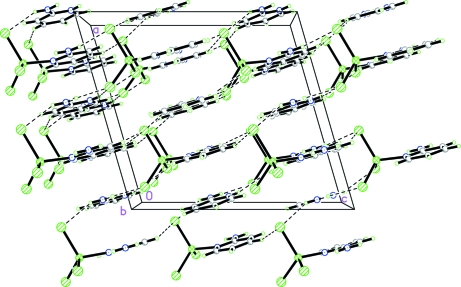
The crystal packing of the title compound, viewed along the b axis. Hydrogen bonds are shown as dashed lines.

## supplementary crystallographic information

### Comment

2-Aminopyridine is used in the manufacture of pharmaceuticals, especially antihistaminic drugs (Windholz, 1976). As a part of our investigations on the binding modes of 2-aminopyridine with metals, we report here the crystal structure of 2-aminopyridinium (2-aminopyridine)trichloronickel(II).

The asymmetric unit of the title compound contains one 2-aminopyridinium cation and one (2-aminopyridine)trichloronickel(II) anion. Protonation of atom N3 of the uncomplexed 2-aminopyridine results in the widening of the C6—N3—C10 angle to 123.3 (2)°, which is 117.7 (1)° in neutral 2-aminopyridine (Chao *et al.*, 1975). The bond lengths and angles are comparable with those observed in related structures (Bis & Zaworotko, 2005; Smith *et al.*, 2000; Jebas *et al.*, 2006).

In the monomeric complex, the Ni^II^ ion is four-coordinated by three Cl anions and the N atom of the 2-aminopyridine ligand, forming a distorted tetrahedral coordination (Fig 1). The Ni—Cl bond lengths (Table 1) are comparable with that reported in the literature (Valdés-Martínez *et al.*, 2001; Batsanov *et al.*, 2001; Sletten & Kovacs, 1993; Corain *et al.*, 1985; Stibrany *et al.*, 2004). The Cl—Ni—Cl bond angles (107.77 (2)° and 108.62 (2)°) are close to the values reported in the literature (Smith *et al.*, 2001; Wei *et al.*, 1995). The dihedral angle between the pyridine and pyridinium rings is 0.9 (2)°.

In the crystal structure, the cations and anionic complexes are stacked into chains along the a, b and c axes and are linked into a three-dimensional framework by N—H···Cl hydrogen bonds (Fig 2).

### Experimental

Solutions of 2-aminopyridine and NiCl_2_.2H_2_O in water were mixed in a molar ratio of 2:1. Few drops of dilute hydrochloric acid were added to the solution and heated at 363 K for 2 h. Blue crystals of the title compound were obtained by slow evaporation after a period of one week.

### Refinement

After checking their presence in a difference map, all H atoms except H1N3 were placed in calculated positions, with C—H = 0.93 Å and N—H = 0.86 Å and refined using a riding model, with *U*_iso_(H) = 1.2*U*_eq_(C,*N*). Atom H1N3 was refined isotropically.

### Crystal data

**Table:**

(C_5_H_7_N_2_)[NiCl_3_(C_5_H_6_N_2_)]	*F*_000_ = 720
*M**_r_* = 354.3	*D*_x_ = 1.68 Mg m^−^^3^
Monoclinic, *C**c*	Mo *K*α radiation λ = 0.71073 Å
Hall symbol: C -2yc	Cell parameters from 8411 reflections
*a* = 12.9265 (1) Å	θ = 3.0–30.6º
*b* = 8.0644 (1) Å	µ = 1.94 mm^−^^1^
*c* = 13.9893 (1) Å	*T* = 100.0 (1) K
β = 106.163 (1)º	Block, blue
*V* = 1400.67 (2) Å^3^	0.37 × 0.08 × 0.07 mm
*Z* = 4	

### Data collection

**Table:**

Bruker SMART APEXII CCD area-detector diffractometer	5088 reflections with *I* > 2σ(*I*)
Detector resolution: 8.33 pixels mm^-1^	*R*_int_ = 0.031
ω scans	θ_max_ = 40.6º
Absorption correction: multi-scan(SADABS; Bruker, 2005)	θ_min_ = 3.0º
*T*_min_ = 0.533, *T*_max_ = 0.876	*h* = −23→23
19539 measured reflections	*k* = −14→14
6427 independent reflections	*l* = −25→16

### Refinement

**Table:**

Refinement on *F*^2^	H atoms treated by a mixture of independent and constrained refinement
Least-squares matrix: full	*w* = 1/[σ^2^(*F*_o_^2^) + (0.034*P*)^2^] where *P* = (*F*_o_^2^ + 2*F*_c_^2^)/3
*R*[*F*^2^ > 2σ(*F*^2^)] = 0.035	(Δ/σ)_max_ < 0.001
*wR*(*F*^2^) = 0.079	Δρ_max_ = 0.52 e Å^−^^3^
*S* = 1.05	Δρ_min_ = −0.64 e Å^−^^3^
6427 reflections	Extinction correction: none
167 parameters	Absolute structure: Flack (1983), 1953 Friedel pairs
2 restraints	Flack parameter: 0.065 (9)

### Special details

**Table:**

Geometry. All e.s.d.'s (except the e.s.d. in the dihedral angle between two l.s. planes) are estimated using the full covariance matrix. The cell e.s.d.'s are taken into account individually in the estimation of e.s.d.'s in distances, angles and torsion angles; correlations between e.s.d.'s in cell parameters are only used when they are defined by crystal symmetry. An approximate (isotropic) treatment of cell e.s.d.'s is used for estimating e.s.d.'s involving l.s. planes.

### Fractional atomic coordinates and isotropic or equivalent isotropic displacement parameters (Å^2^)

**Table:**

	*x*	*y*	*z*	*U*_iso_*/*U*_eq_	
Ni1	0.245028 (18)	0.65589 (3)	0.188212 (18)	0.01865 (6)	
Cl1	0.40142 (4)	0.66552 (7)	0.14504 (4)	0.01889 (9)	
Cl2	0.19741 (4)	0.38637 (6)	0.19116 (4)	0.02255 (10)	
Cl3	0.10912 (4)	0.79387 (7)	0.07906 (4)	0.02299 (10)	
N1	0.26459 (13)	0.7760 (2)	0.31947 (12)	0.0157 (3)	
N2	0.30301 (15)	0.5422 (2)	0.41934 (14)	0.0228 (4)	
H2B	0.2895	0.4816	0.3667	0.027*	
H2C	0.322	0.4966	0.4772	0.027*	
N3	0.55299 (14)	0.0900 (2)	0.44965 (14)	0.0191 (3)	
N4	0.53731 (16)	−0.1509 (2)	0.35719 (15)	0.0228 (4)	
H4B	0.5574	−0.2072	0.4114	0.027*	
H4C	0.5224	−0.2007	0.3006	0.027*	
C1	0.29457 (15)	0.7074 (3)	0.41122 (15)	0.0181 (4)	
C2	0.31669 (17)	0.8074 (3)	0.49792 (16)	0.0213 (4)	
H2A	0.3384	0.7591	0.5607	0.026*	
C3	0.30568 (17)	0.9761 (3)	0.48792 (18)	0.0254 (4)	
H3A	0.3199	1.0431	0.5442	0.03*	
C4	0.27352 (17)	1.0463 (3)	0.39438 (18)	0.0245 (4)	
H4A	0.2652	1.1605	0.3867	0.029*	
C5	0.25411 (16)	0.9441 (3)	0.31322 (17)	0.0198 (4)	
H5A	0.2326	0.992	0.2503	0.024*	
C6	0.52944 (15)	0.0123 (2)	0.36071 (15)	0.0173 (3)	
C7	0.49768 (16)	0.1116 (3)	0.27449 (16)	0.0203 (4)	
H7A	0.4813	0.0628	0.2118	0.024*	
C8	0.49108 (16)	0.2795 (3)	0.28322 (16)	0.0217 (4)	
H8A	0.4704	0.3447	0.2262	0.026*	
C9	0.51508 (17)	0.3550 (3)	0.37731 (18)	0.0229 (4)	
H9A	0.5093	0.4693	0.3832	0.027*	
C10	0.54691 (16)	0.2575 (3)	0.45963 (17)	0.0222 (4)	
H10A	0.5645	0.3051	0.5227	0.027*	
H1N3	0.571 (2)	0.030 (3)	0.499 (2)	0.023 (7)*	

### Atomic displacement parameters (Å^2^)

**Table:**

	*U*^11^	*U*^22^	*U*^33^	*U*^12^	*U*^13^	*U*^23^
Ni1	0.02016 (11)	0.02167 (12)	0.01419 (12)	−0.00140 (10)	0.00489 (9)	−0.00168 (10)
Cl1	0.0188 (2)	0.0211 (2)	0.0179 (2)	−0.00022 (15)	0.00699 (18)	−0.00025 (16)
Cl2	0.0285 (2)	0.0194 (2)	0.0211 (2)	−0.00610 (19)	0.0092 (2)	−0.00511 (18)
Cl3	0.0208 (2)	0.0324 (3)	0.0142 (2)	0.00279 (19)	0.00214 (17)	0.00196 (19)
N1	0.0151 (7)	0.0181 (7)	0.0133 (7)	−0.0002 (6)	0.0030 (6)	0.0000 (6)
N2	0.0313 (9)	0.0201 (9)	0.0150 (8)	0.0021 (7)	0.0032 (7)	0.0003 (6)
N3	0.0170 (7)	0.0273 (9)	0.0132 (8)	0.0013 (6)	0.0043 (6)	0.0020 (7)
N4	0.0283 (9)	0.0220 (8)	0.0168 (9)	−0.0019 (7)	0.0038 (7)	0.0029 (6)
C1	0.0146 (8)	0.0238 (10)	0.0165 (9)	0.0012 (6)	0.0053 (7)	0.0011 (7)
C2	0.0180 (8)	0.0307 (11)	0.0149 (9)	−0.0009 (7)	0.0043 (7)	−0.0018 (8)
C3	0.0224 (9)	0.0269 (11)	0.0278 (12)	−0.0030 (8)	0.0087 (9)	−0.0105 (9)
C4	0.0251 (10)	0.0189 (9)	0.0309 (12)	−0.0026 (8)	0.0099 (9)	−0.0067 (9)
C5	0.0195 (8)	0.0179 (9)	0.0224 (10)	−0.0007 (7)	0.0065 (8)	−0.0002 (8)
C6	0.0153 (8)	0.0220 (9)	0.0142 (9)	−0.0021 (7)	0.0035 (7)	0.0011 (7)
C7	0.0183 (8)	0.0269 (10)	0.0145 (9)	0.0001 (7)	0.0023 (7)	0.0026 (8)
C8	0.0201 (9)	0.0261 (10)	0.0185 (10)	0.0034 (8)	0.0050 (8)	0.0059 (8)
C9	0.0204 (9)	0.0225 (10)	0.0272 (12)	0.0019 (7)	0.0092 (9)	−0.0010 (8)
C10	0.0183 (8)	0.0288 (11)	0.0200 (10)	0.0008 (8)	0.0062 (8)	−0.0052 (8)

### Geometric parameters (Å, °)

**Table:**

Ni1—N1	2.0287 (17)	C2—C3	1.371 (3)
Ni1—Cl2	2.2625 (6)	C2—H2A	0.93
Ni1—Cl1	2.2665 (5)	C3—C4	1.380 (3)
Ni1—Cl3	2.2722 (6)	C3—H3A	0.93
N1—C1	1.352 (3)	C4—C5	1.369 (3)
N1—C5	1.363 (3)	C4—H4A	0.93
N2—C1	1.339 (3)	C5—H5A	0.93
N2—H2B	0.86	C6—C7	1.410 (3)
N2—H2C	0.86	C7—C8	1.364 (3)
N3—C6	1.350 (3)	C7—H7A	0.93
N3—C10	1.362 (3)	C8—C9	1.404 (3)
N3—H1N3	0.82 (3)	C8—H8A	0.93
N4—C6	1.322 (3)	C9—C10	1.360 (3)
N4—H4B	0.86	C9—H9A	0.93
N4—H4C	0.86	C10—H10A	0.93
C1—C2	1.417 (3)		
			
N1—Ni1—Cl2	114.10 (5)	C2—C3—C4	120.0 (2)
N1—Ni1—Cl1	109.21 (5)	C2—C3—H3A	120
Cl2—Ni1—Cl1	107.77 (2)	C4—C3—H3A	120
N1—Ni1—Cl3	104.63 (5)	C5—C4—C3	118.5 (2)
Cl2—Ni1—Cl3	108.62 (2)	C5—C4—H4A	120.8
Cl1—Ni1—Cl3	112.60 (2)	C3—C4—H4A	120.8
C1—N1—C5	117.72 (18)	N1—C5—C4	123.6 (2)
C1—N1—Ni1	126.48 (14)	N1—C5—H5A	118.2
C5—N1—Ni1	115.59 (13)	C4—C5—H5A	118.2
C1—N2—H2B	120	N4—C6—N3	119.8 (2)
C1—N2—H2C	120	N4—C6—C7	122.7 (2)
H2B—N2—H2C	120	N3—C6—C7	117.51 (19)
C6—N3—C10	123.36 (19)	C8—C7—C6	119.8 (2)
C6—N3—H1N3	115.9 (18)	C8—C7—H7A	120.1
C10—N3—H1N3	120.7 (18)	C6—C7—H7A	120.1
C6—N4—H4B	120	C7—C8—C9	120.7 (2)
C6—N4—H4C	120	C7—C8—H8A	119.6
H4B—N4—H4C	120	C9—C8—H8A	119.6
N2—C1—N1	118.88 (18)	C10—C9—C8	118.6 (2)
N2—C1—C2	120.05 (19)	C10—C9—H9A	120.7
N1—C1—C2	121.1 (2)	C8—C9—H9A	120.7
C3—C2—C1	119.1 (2)	C9—C10—N3	119.9 (2)
C3—C2—H2A	120.4	C9—C10—H10A	120
C1—C2—H2A	120.4	N3—C10—H10A	120
			
Cl2—Ni1—N1—C1	28.37 (17)	C2—C3—C4—C5	−0.5 (3)
Cl1—Ni1—N1—C1	−92.28 (15)	C1—N1—C5—C4	0.8 (3)
Cl3—Ni1—N1—C1	146.95 (15)	Ni1—N1—C5—C4	−174.29 (16)
Cl2—Ni1—N1—C5	−156.98 (11)	C3—C4—C5—N1	0.1 (3)
Cl1—Ni1—N1—C5	82.36 (13)	C10—N3—C6—N4	179.99 (18)
Cl3—Ni1—N1—C5	−38.41 (13)	C10—N3—C6—C7	0.4 (3)
C5—N1—C1—N2	178.47 (16)	N4—C6—C7—C8	179.96 (19)
Ni1—N1—C1—N2	−7.0 (3)	N3—C6—C7—C8	−0.5 (3)
C5—N1—C1—C2	−1.4 (3)	C6—C7—C8—C9	−0.3 (3)
Ni1—N1—C1—C2	173.14 (13)	C7—C8—C9—C10	1.1 (3)
N2—C1—C2—C3	−178.84 (18)	C8—C9—C10—N3	−1.2 (3)
N1—C1—C2—C3	1.0 (3)	C6—N3—C10—C9	0.5 (3)
C1—C2—C3—C4	0.0 (3)		

### Hydrogen-bond geometry (Å, °)

**Table:**

*D*—H···*A*	*D*—H	H···*A*	*D*···*A*	*D*—H···*A*
N3—H1N3···Cl2^i^	0.82 (3)	2.81 (3)	3.380 (2)	128 (2)
N2—H2B···Cl2	0.86	2.53	3.3475 (19)	159
N2—H2C···Cl1^ii^	0.86	2.63	3.4866 (19)	172
N4—H4B···Cl3^i^	0.86	2.36	3.197 (2)	165
N4—H4C···Cl1^iii^	0.86	2.54	3.344 (2)	156

Symmetry codes: (i) *x*+1/2, −*y*+1/2, *z*+1/2; (ii) *x*, −*y*+1, *z*+1/2; (iii) *x*, *y*−1, *z*.
